# Interventions to reduce short-wavelength (“blue”) light exposure at night and their effects on sleep: A systematic review and meta-analysis

**DOI:** 10.1093/sleepadvances/zpaa002

**Published:** 2020-06-04

**Authors:** Ari Shechter, Kristal A Quispe, Jennifer S Mizhquiri Barbecho, Cody Slater, Louise Falzon

**Affiliations:** Center for Behavioral Cardiovascular Health, Columbia University Irving Medical Center, New York, NY; Sleep Center of Excellence, Columbia University Irving Medical Center, New York, NY; Center for Behavioral Cardiovascular Health, Columbia University Irving Medical Center, New York, NY; Center for Behavioral Cardiovascular Health, Columbia University Irving Medical Center, New York, NY; Columbia University Vagelos College of Physicians and Surgeons, New York, NY; Center for Personalized Health, Feinstein Institutes for Medical Research, Northwell Health, New York, NY

**Keywords:** sleep, circadian, blue light, short wavelength, intervention, systematic review, meta-analysis

## Abstract

The sleep-wake and circadian cycles are influenced by light, particularly in the short-wavelength portion of the visible spectrum. Most personal light-emitting electronic devices are enriched in this so-called “blue” light. Exposure to these devices in the evening can disturb sleep. Interventions to reduce short-wavelength light exposure before bedtime may reduce adverse effects on sleep. We conducted a systematic review and meta-analysis to examine the effect of wearing color-tinted lenses (e.g. orange or amber) in frames to filter short-wavelength light exposure to the eye before nocturnal sleep. Outcomes were self-reported or objective measures of nocturnal sleep. Relatively few (*k* = 12) studies have been done. Study findings were inconsistent, with some showing benefit and others showing no effect of intervention. Meta-analyses yielded a small-to-medium magnitude combined effect size for sleep efficiency (Hedge’s *g* = 0.31; 95% CI: −0.05, 0.66; *I*^2^ = 38.16%; *k* = 7), and a small-to-medium combined effect size for total sleep time (Hedge’s *g* = 0.32; 95% CI: 0.01, 0.63; *I*^2^ = 12.07%; *k* = 6). For self-report measures, meta-analysis yielded a large magnitude combined effects size for Pittsburgh Sleep Quality Index ratings (Hedge’s *g* = −1.25; 95% CI: −2.39, −0.11; *I*^2^ = 36.35%; *k* = 3) and a medium combined effect size for total sleep time (Hedge’s *g* = 0.51; 95% CI: 0.18, 0.84; *I*^2^ = 0%; *k* = 3), Overall, there is some, albeit mixed, evidence that this approach can improve sleep, particularly in individuals with insomnia, bipolar disorder, delayed sleep phase syndrome, or attention-deficit hyperactive disorder. Considering the ubiquitousness of short-wavelength-enriched light sources, future controlled studies to examine the efficacy of this approach to improve sleep are warranted.

Systematic review registration: PROSPERO 2018 CRD42018105854.

**Statement of Significance**

The sleep-wake cycle is influenced by light exposure, particularly short-wavelength light of ~460 nm. Portable light-emitting electronic devices are typically enriched in short-wavelength light; accordingly, use of these devices before bedtime can disturb sleep initiation, maintenance, and quality. Approaches to selectively reduce or filter short-wavelength light exposure to the eyes in the hours preceding bedtime, while allowing for maintained use of light-emitting electronic devices, may ameliorate adverse effects of so-called “blue” light on sleep. To our knowledge, this is the first systematic review and meta-analysis to examine the effect of wearing color-tinted lenses (e.g. orange or amber) in frames before bedtime on nocturnal sleep quality and duration. We observed that using this approach to reduce exposure to pre-bedtime short-wavelength light via “blue-blocking” glasses could be effective in improving sleep, particularly in individuals susceptible to sleep disturbances and/or with psychiatric disorders.

## Introduction

In humans, the homeostatic regulation of sleep interacts with the circadian system to foster a consolidated nocturnal sleep episode that typically coincides with ambient darkness and increased circulating melatonin levels [1–3]. Melatonin, under circadian control, is a hormonal signal of the biological night, and a factor that “opens the sleep gate” to initiate the nocturnal sleep period [4]. In addition to its endogenous regulation, the circadian system is highly sensitive to external light. The circadian photoreceptor system, via intrinsically photosensitive retinal ganglion cells, shows peak sensitivity to ~450–480 nm light within the short-wavelength portion of the visible spectrum [5, 6]. This peak sensitivity of the circadian photoreceptor system to so-called “blue light” accounts for the high efficacy of light within this spectral range to suppress melatonin secretion and increase neurocognitive alertness [7].

This has practical ramifications for sleep and circadian physiology since most smartphones, televisions, computers, and increasingly, domestic light bulbs, are lit by light-emitting diodes (LED) sources that are enriched in short-wavelength light of ~460 nm [8]. Recent work has demonstrated that evening exposure to LED-backlit computers and self-luminous personal devices such as tablets can suppress and delay melatonin secretion, decrease sleepiness, prolong sleep initiation, and worsen sleep quality [8–13].

The use of personal light-emitting electronic devices is nearly ubiquitous, with most people reporting continued use within the hour before bedtime [14]. Accordingly, huge portions of the population are engaging in behaviors that can potentially worsen sleep initiation and quality [15]. Individuals are unlikely to limit use of such devices, however, even when recommendations are made by clinicians. Novel approaches to minimize the effects of nocturnal blue light exposure, while allowing for the maintained use of these devices, may, therefore, have widespread benefits for sleep health.

Interventions aimed at selectively reducing or filtering out short-wavelength light exposure to the eyes in the hours preceding bedtime may ameliorate adverse effects of light on sleep. These approaches may include wearing amber or orange-colored “blue light-blocking” spectacles. To our knowledge, no systematic review and meta-analysis have yet been conducted to describe the evidence and efficacy of this approach. Our goal was to examine the effect of this intervention aimed at reducing retinal exposure to short-wavelength light in the hours before bedtime, via “blue-blocking” spectacles, on sleep initiation, quality, and duration.

## Methods

### Search strategy

This systematic review was conducted under the guidance of Preferred Reporting Items for Systematic Reviews and Meta-Analyses (PRISMA) criteria [16]. The systematic review was registered on PROSPERO, the international prospective register of systematic reviews (PROSPERO 2018 CRD42018105854). A literature search of six biomedical electronic databases (MEDLINE, EMBASE, The Cochrane Library, PsycINFO, CINAHL, and AMED) was conducted from database inception to November 2019. All relevant subject headings and free-text terms were used to represent sleep and short-wavelength light. Ongoing studies were also sought through clinicaltrials.gov and the WHO International Clinical Trials Registry Platform. Additional records were identified by employing the Similar Articles feature in PubMed, manually searching the reference lists of relevant studies to identify relevant references missed by automated searches, and conducting a search of gray literature to identify studies not indexed in the databases listed above. There was no restriction on publication period or language of publication. The full search strategy is available as Supplementary Material.

### Study selection

The primary criterion to be eligible for inclusion in the current review was that the study administered the intervention aimed at reducing, blocking, or selectively filtering nocturnal blue-wavelength light exposure to the eye. Specifically, the intervention approach was the use of short-wavelength light-blocking glasses or spectacles (amber-, orange-, brown-, or yellow-colored lenses worn in frames over the eyes, i.e., “blue blockers”). Further inclusion criteria of the intervention were that (1) the use of the intervention occurred in the hours before nocturnal bedtime and (2) the goal of the intervention was to improve sleep (duration, quality, initiation, etc.). In addition to the intervention criterion, included studies assessed at least one relevant sleep outcome (objective or subjective). Both healthy and pathological populations were included; i.e. having a medical, psychiatric, sleep, or circadian rhythms disorder was not exclusionary. There was no restriction on the setting of the research: interventions could have been administered at home, in the laboratory, in hospital/clinical settings, or in a combination of these. Studies could have included individuals of all ages and sexes. Experimental designs could have been parallel-arm or crossover randomized controlled trials (RCTs), open-label single-group studies, or before-after designs. Comparator groups varied depending on study design, but could have included (1) sham control (either as a separate group or a crossover condition) or (2) baseline pre-treatment levels (compared to post-treatment).

Studies were excluded from the review if they did not administer the relevant intervention or report a relevant outcome. In addition, studies were excluded if they administered the intervention during the daytime. Studies on individuals who were shift workers were not included. Although short-wavelength light-blocking approaches are sometimes used in this population [17], timing of administration is different from those with a nocturnal sleep episode, in an attempt to improve daytime sleep episode, or alter circadian physiology to foster a daytime sleep episode. Some work has been done to test the effects of short-wavelength light-blocking during the daytime on eyestrain, eye fatigue, visual performance, macular integrity, etc [18]. Since this approach blocks short-wavelength light in the daytime and has different outcomes of interest (i.e. is not related to nocturnal sleep propensity, quality, and/or duration), these studies were not included. Related to this, some studies considered the effects of cataract surgery or implanted short-wavelength-light filtering intraocular lenses [19, 20]. These types of studies were also excluded since they do not meet the criterion of selective blockage of short-wavelength light in the period before nocturnal sleep.

**Figure 1.**
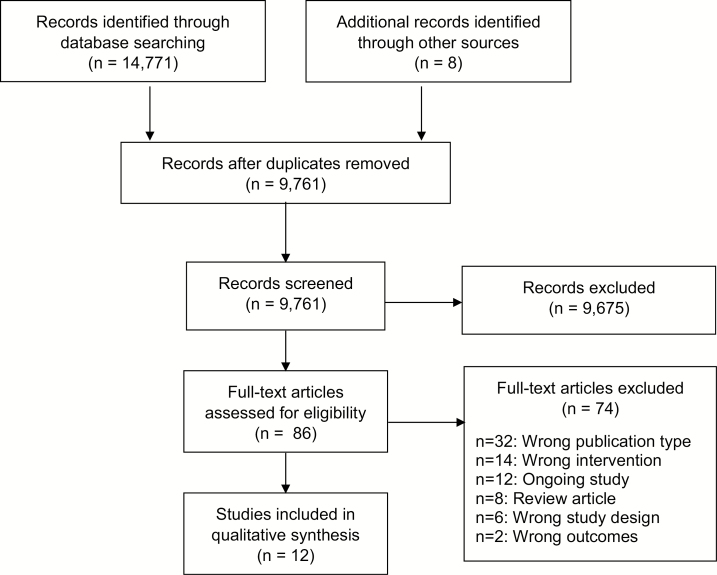
Flow chart for selection of references included in systematic review.

Individual titles, abstracts, and full-text articles were independently screened by two separate investigators using the inclusion and exclusion criteria outlined above. Where disagreements occurred, a consensus meeting among investigators was held to come to a decision about study inclusion. A PRISMA flow diagram summarizes included and excluded studies and the reasons for their exclusion (Figure 1).

### Data extraction

All obtained references were reviewed, and if retained, data extraction was conducted independently by two investigators. Key data extracted included: title of study, first author, year of publication, study sample size, participant age and sex, study method/design, intervention details, description of control group, duration of trial, methods to assess sleep outcomes, outcome variables, and group differences. When available, information on transmission profile of short-wavelength light (% blue-light transmittance [BLT]) and visible light (visible light transmittance [VLT]), or the transition points (e.g. % light blocked below certain wavelengths) is included in the intervention characteristics. Discrepancies in data extraction were resolved by a third investigator and consensus. If outcomes were likely collected but not reported in the published paper (i.e. specific accelerometry parameters like sleep onset latency [SOL] or wake after sleep onset [WASO]), the corresponding author of the paper was contacted and a request for the data was made.

### Risk of bias assessment

Studies were classified as low-, high-, or unclear risk of bias by two investigators. Risk of bias assessments were based on the Cochrane Risk of Bias Assessment Tool for RCTs [21] or the Cochrane Effective Practice and Organisation of Care (EPOC) Suggested Risk of Bias Criteria for EPOC Reviews for non-randomized (e.g. before-after studies) studies. For RCTs, risk of bias was assessed in the following seven criteria: selection bias (improper sequence generation or allocation concealment), performance bias (lack of blinding of participants or personnel to sleep/circadian outcomes), detection bias (blinding of assessors of sleep/circadian outcomes), attrition bias (incomplete outcome data), selective outcome reporting, and other bias (groups different at baseline). For non-randomized studies, risk of bias was assessed in the following eight criteria: selection bias (improper sequence generation or allocation concealment), baseline measures and characteristics similar, attrition bias, knowledge of intervention prevented, protection against contamination, and selective outcome reporting.

### Meta-analysis

Combined effects sizes were estimated with Hedge’s *g* based on reported means and SDs, and were computed using a random-effects model. Subgroup analyses were conducted with groups “healthy participants” and “sleep and/or psychiatric disorder,” using a random-effects model. Effects size heterogeneity was estimated with *I*^2^. If excessive heterogeneity of effect sizes were observed (*I*^2^ > 75%), individual effect sizes from studies were not combined to yield a pooled estimate. Meta-analyses were conducted when data were available on a given outcome from a minimum of three studies. Analyses were completed with the Meta-Essentials tool (version 1.4) [22].

## Results

### Literature search

A total of 14,771 references were identified through database searching, with eight more found as additional records identified through other sources. There were 9,761 references after de-duplication. After screening titles and abstracts, 86 articles were selected for full-text review. Twelve studies were ultimately included in the systematic review (Figure 1).

### Characteristics of included studies

Among the 12 included studies, eight were RCTs, and four were before-after studies (Table 1). The analyzed sample sizes were generally small and ranged from 6 to 21 participants. The mean ages of participants ranged from 16.5 to 48.8 years. Percentage of female participants ranged from 0% to 80% of the samples. Characteristics of participants varied and included four studies in healthy adults [23–26], one study in healthy adolescents [27], three studies in individuals with insomnia/sleep difficulties [28–30], one study in individuals with major depressive disorder (MDD) and sleep-onset insomnia [31], one study in individuals with delayed sleep phase syndrome (DSPS) [32], one study in individuals with attention-deficit hyperactive disorder (ADHD) and DSPS [33], and one study in individuals hospitalized with bipolar disorder in a manic state [34]. Duration of interventions ranged from 2 days to 1 month (Table 1). Among the 12 studies, four assessed sleep with only self-report measures [24, 28, 31, 33], three assessed sleep with objective measures [30, 32, 34], and five assessed sleep with both self-report and objective measures [23, 25–27, 29] (Table 1).

**Table 1.** Design details of all included studies

Author, year (ref #)	Study design	Participants	Sample size	Age, year (SD)	Sex, female (%)	Duration	Intervention details (lenses transmission)	Control details (lenses transmission)	Outcomes methods
Ayaki 2016 [25]	Sham-controlled, crossover; RCT	Healthy adults	*n* = 12	29.0 (5.0)	50	2 days	Brown lenses (43.8% BLT, 76.9% VLT) worn 10:00 pm to 12:00 am, while using portable luminous devices	Gray lenses (93.2% BLT, 76.4% VLT)	Accelerometry sleep Self-report sleep
Burkhart 2009 [28]	Sham-controlled, parallel; RCT	Sleep difficulty, adults	*n* = 10 Tx *n* = 10 Cx	34.0 (8.2)	55	2 weeks	Amber lenses (~100% of <550 nm light blocked) worn 3 hours before bedtime	Yellow lenses	Self-report sleep
Esaki 2016 [32]	Baseline to post-Tx	DSPS adults	*n* = 7	18.1 (3.18)	22	3 weeks	Amber lenses (~100% of <530 nm light blocked) worn 09:00 pm to bedtime	NA	Accelerometry sleep
Esaki 2017 [31]	Sham-controlled, parallel; RCT	MDD and sleep onset insomnia, adults	*n* = 9 Tx *n* = 8 Cx	Tx: 43.4 (8.4) Cx: 39.8 (5.8)	70	2 weeks	Orange lenses worn 08:00 pm to bedtime (~100% of <530 nm light blocked)	Clear lenses	Self-report sleep
Fargason 2013 [33]	Baseline to post-Tx	ADHD and DSPS, adults	*n* = 14	43.9 (range: 21–76)	57	2 weeks	Amber lenses (~100% of <530 nm light absorbed) worn at least 3 hours before turning off ambient light	NA	Self-report sleep
Henriksen 2020 [34]	Sham-controlled, parallel; RCT	Bipolar-manic, adults	*n* = 10 Tx *n* = 10 Cx	Tx: 43.9 (11.8) Cx: 48.8 (14.1)	30	7 days	Orange lenses worn 06:00 pm to 08:00 pm, removed for sleep (transmission not provided)	Clear lenses	Accelerometry sleep
Knufinke 2019 [23]	Sham-controlled, crossover; RCT	Healthy adults	*n* = 15	23.27 (3.63)	80	7 days	Amber lenses (~100% of <400 nm light blocked, and 89–99.9% of 400–500 nm light blocked) worn 3 hours before bedtime	Clear lenses	Accelerometry sleep Self-report sleep
Nagai 2019 [24]	Baseline to post-Tx	Healthy adults	*n* = 6	41.7 (6.8)	50	1 month	Brown lenses(~100% of 395–490 nm light blocked; 30% VLT) worn 2–3 hours before bedtime	N/A	Self-report sleep
Ostrin 2017 [26]	Baseline to post-Tx	Healthy adults	*n* = 21	26.7 (7.8)	48	2 weeks	Orange lenses worn 08:00 pm to bedtime or for at least 3 hours before bedtime (~99% of <540 nm light absorbed; ~90% of >540 nm light transmitted)	N/A	Accelerometry sleep Self-report sleep
Perez Algorta 2018 [30]	Sham-controlled, crossover; RCT	Sleep difficulty, adults	*n* = 12	18.5 (0.52)	67	4 days	Amber lenses (35% BLT, 90% VLT) worn 3 hours before bedtime	Blue lenses (57% VLT)	Peripheral arterial tone plus pulse oximetry for sleep
Shechter 2018 [29]	Sham-controlled, crossover; RCT	Insomnia adults	*n* = 14	46.6 (11.5)	57	7 days	Amber lenses (35% BLT, 90% VLT) worn 2 hours before bedtime	Clear lenses (90% BLT, 92% VLT)	Accelerometry sleep Self-report sleep
van der Lely 2015 [27]	Sham-controlled, crossover; RCT	Healthy adolescents	*n* = 13	16.5 (0.66)	0	7 days	Orange lenses (1.8% BLT, 30% VLT) worn 06:00 pm to bedtime during the ambulatory portion of the study, and in the laboratory portion of the study, for three hours preceding bedtime while exposed to LED	Clear lenses (91.8% BLT, 92% VLT)	Accelerometry sleep PSG sleep Self-report sleep

ADHD: Attention-deficit hyperactive disorder; BB: Blue blocker; BLT: Blue-light transmission; Cx: Control group; DSPS: Delayed sleep phase syndrome; MDD: Major depressive disorder; NA: Not applicable; NR: Not reported; PSG: Polysomnography; RCT: Randomized controlled trial; Tx: Treatment group; VLT: Visible light transmission.

### Intervention characteristics

Interventions that used the short-wavelength light blocking (i.e. “blue-blocking”, BB) lenses-in-frames approach in the evening preceding the nocturnal sleep episode and that assessed a sleep outcome are described below (Table 1).

#### Healthy individuals.

In the Ayaki *et al.* study, healthy adults wore lenses from 10:00 pm to 12:00 am before bedtime while using portable luminous devices. Lenses were brown-tinted with a 43.8% BLT and 76.9% VLT. The (sham) control condition was gray-tinted lenses with 93.2% BLT and 76.4% VLT. The intervention was administered for 2 consecutive days in a crossover design [25]. In the Ostrin *et al.* study, healthy adults wore lenses from 10:00 pm to bedtime, or from 3 hours before bedtime if habitual bedtime was before 11:00 pm. The orange lenses absorbed ~99% of light <540 nm and transmitted ~90% of light longer than 540 nm. Participants wore the lenses for 2 weeks, and outcomes were compared after treatment to pre-treatment baseline [26]. The Knufinke *et al.* [23] study included healthy adults who were recreational athletes and who reported good sleep quality. Participants wore BB lenses or clear sham control lenses for 7 days for 3 hours preceding bedtime, in a randomized crossover design. The amber lenses completely blocked wavelengths <400 nm and blocked 89–99.9% of wavelengths between 400 and 500 nm [23]. In the Nagai *et al.* [24] study, six healthy adults wore BB lenses for 2–3 hours preceding bedtime. The lenses were brown-tinted and blocked wavelengths between 395 and 490 nm, with 30% VLT. The lenses were worn each night for 1 month and compared to pre-treatment baseline. In the van der Lely study, healthy adolescents, who reported spending on average more than 2.5 hours per evening in front of media screens, wore orange-tinted or clear lenses in frames from 06:00 pm until sleep onset, during an ambulatory week, and in the laboratory while being exposed to a 3-hour LED computer screen with 482 nm peak wavelength [27]. The BB lenses had 98.2% blue light absorption (BLA) and 30% VLT and the clear lenses had 91.8% BLT and 92% VLT. This was a counterbalanced crossover design, with a 1- to 5-week washout period between treatment phases.

#### Individuals with sleep disturbance.

In the Burkhart and Phelps study, participants who had sleep complaints wore amber-tinted lenses (blocking wavelengths <550 nm) in glasses for 3 hours preceding their normal bedtime [28]. The intervention was administered for 2 weeks and compared to pre-treatment baseline and control condition, and also to a control lenses condition. In the Shechter *et al.* study, individuals with insomnia symptoms wore lenses for 2 hours before bedtime for seven nights in active and sham conditions, in a randomized crossover design. Active lenses were amber-tinted with a 35% BLT and 90% VLT [29]. The sham control condition was clear lenses in frames with 90% BLT and 92% VLT. Experimental phases were separated by a 4-week washout period. In the Esaki *et al.* [32] study, adults with DSPS wore amber glasses (blocking wavelengths < 530 nm) from 09:00 pm to bedtime each night for 3 weeks. Comparisons were made to pre-treatment baseline with no lenses. In the Perez Algorta *et al.* [30] study, first-year undergraduate students with sleep complaints wore amber-tinted lenses or blue-colored lenses (i.e. non-short-wavelength blocking control) in glasses for 3 hours preceding bedtime. The intervention was administered for four nights in each condition, in a randomized crossover design. There was a 3-day (no glasses) washout between treatment phases.

#### Individuals with psychiatric conditions.

A second study by Esaki *et al.* tested the effects of wearing orange lenses in frames (blocking wavelengths below approximately 530 nm), compared to clear glasses sham control group in a parallel-arm RCT. Lenses were worn from 08:00 pm to bedtime each night for 2 weeks in individuals with MDD and sleep onset insomnia [31]. In the Fargason *et al.* study, individuals with ADHD and DSPS wore amber glasses (blocking wavelengths < 530 nm) from sundown until bedtime every night, with a minimum of 3 hours prior to time of lights off [33]. The lenses were worn for 2 weeks and compared to pre-treatment baseline. In Henriksen *et al.*, hospitalized patients with bipolar disorder in a manic state wore orange-tinted glasses (blocking wavelengths less than approximately 530 nm) from 06:00 pm to 08:00 am (removed at lights-off but put back on if awakening before 08:00 am) for a seven-night period, in a randomized parallel-arm study [34]. Comparisons of sleep actigraphic parameters were made to a clear lenses control condition on the fifth night of treatments.

### Risk of bias assessment

**Figure 2.**
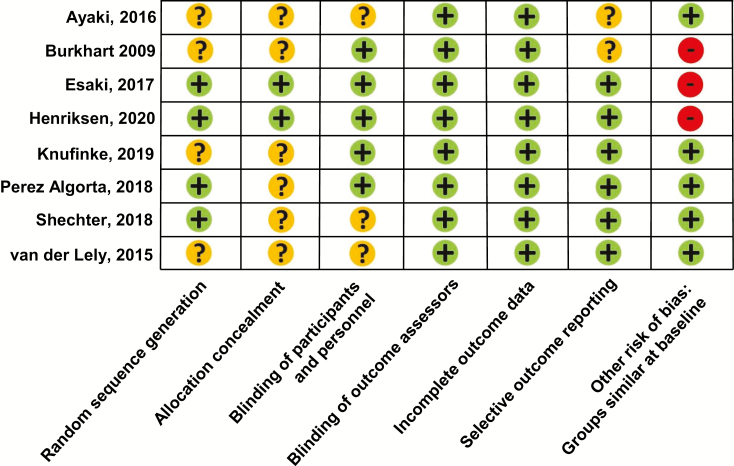
Risk of bias of included randomized controlled trials. Plus signs (green circles) indicate low risk of bias. Minus signs (red circles) indicate high risk of bias. Question marks (yellow circles) indicate unclear risk of bias.

Figure 2 summarizes risk of bias for the included RCTs. Four out of the eight trials (50%) adequately described randomization approaches [29–31, 34]. The other studies, however, state that phases were randomized but do not describe the method of randomization or sequence generation [23, 25, 27, 28]. Most of the studies (75%) did not adequately describe allocation concealment. Most of studies did have adequate blinding of participant/personnel (62.5%) and of outcomes assessors (100%). Studies did not have incomplete outcome data; however, some studies did not report attrition. For most of the studies, the experimental protocol was not published. When there was limited “convincing” text that all pre-specified sleep outcomes were described, the studies were classified as “unclear” for selective outcome reporting (as per the Cochrane recommendations) [21]. In two studies [28, 31], baseline sleep quality characteristics were different between experimental groups, and in the study on individuals with bipolar disorder [34], there were group differences in age and mania ratings, which can potentially confound results.

**Figure 3.**
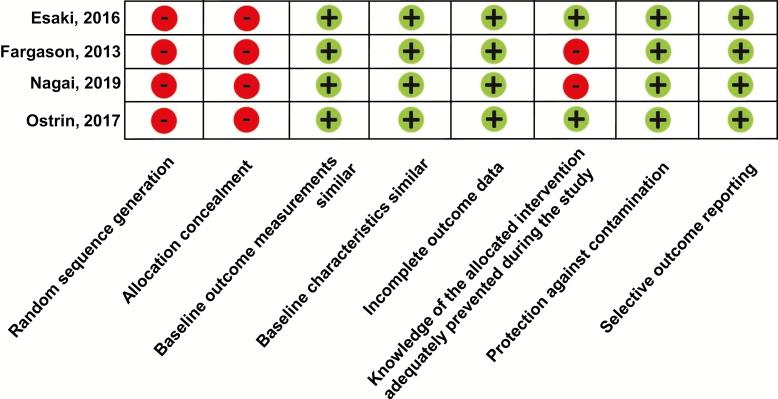
Risk of bias of included before-after studies. Plus signs (green circles) indicate low risk of bias. Minus signs (red circles) indicate high risk of bias. Question marks (yellow circles) indicate unclear risk of bias.

Figure 3 summarizes risk of bias for the non-randomized studies. As per Cochrane EPOC recommendations, since the studies were before-after studies [24, 26, 32, 33], they were deemed to have high risk of selection bias (no random sequence generation). Similarly, the four before-after studies had high risk for selection bias due to no allocation concealment. There was generally low risk of bias on the other components for the non-randomized studies, although two study [24, 33] included only self-report (nonobjective) measures and were thus not blindly assessed.

### Effects of interventions on sleep

#### Objective sleep measures.

Eight studies included objective sleep parameters as an outcome (Table 2). Of these, six used wrist-accelerometry [23, 25, 26, 29, 32, 34], one had both PSG and accelerometer measures [27], and one had peripheral arterial tone plus pulse oximetry [30].

**Table 2.** Effects of interventions on objective sleep

Author, year (ref #)	Sleep assessment method	Findings
Ayaki 2016 [25]	Accelerometry	• SOL was significantly reduced in BB (5.7 ± 2.7 minutes) vs. Cx (13.2 ± 10.8 minutes), *p* < 0.05.
		• SE was significantly increased in BB (97.0 ± 2.8%) vs. Cx (91.8 ± 7.2%), *p* < 0.05.
		• No significant difference in WASO between BB and Cx.
Esaki 2016 [32]	Accelerometry	• Sleep onset time was significantly advanced in BB (0:23 ± 1:38) vs. baseline (2:36 ± 2:18); Change: −2:12, *p* = 0.034.
		• Wake time, TST, WASO*, SOL*, and SE were not significantly different in BB vs. baseline.
Henriksen 2020 [34]	Accelerometry	• SE was significantly increased in BB (92.6%, 95% CI: 89.4–95.8) vs. Cx (83.1%, 95% CI: 75.9–90.3), *p* = 0.027.
		• WASO was significantly reduced in BB (33.5 minutes, 95% CI: 19.8–47.1) vs. Cx (79.2 minutes, 95% CI: 48.0–110.3), *p* = 0.010.
		• No significant difference in TST or sleep fragmentation index in BB vs. Cx.
Knufinke 2019 [23]	Accelerometry	• No significant differences in TST, SE, SOL, or WASO in BB vs. Cx.
Ostrin 2017 [26]	Accelerometry	• TST was increased in BB (431.5 ± 42.9 minutes) vs. baseline (408.7 ± 44.9 minutes), *p* = 0.001.
		• WASO* was increased in BB (56.08 ± 17.0) vs. baseline (49.25 ± 18.43), *p* = 0.030.
		• No significant differences in SE or SOL in BB vs. baseline.
Perez Algorta 2018 [30]	Peripheral arterial tone plus pulse oximetry	• No significant difference in sleep duration, SE, or awakenings in BB vs. Cx.
Shechter 2018 [29]	Accelerometry	• TST was increased in BB (358.80 ± 66.29 minutes) vs. Cx (330.33 ± 66.01 minutes); *p* = 0.035.
		• No significant differences in SE, SOL, or WASO in BB vs. Cx.
van der Lely 2015 [27]	Accelerometry	• No significant difference in TST, SE, fragmentation index in BB vs. Cx.
van der Lely 2015 [27]	Polysomnography	• No significant difference in TST, latency to N1, latency to N2, latency to REM sleep, movement time, WASO, wake after lights out, stage 1%, stage 2%, SWS%, NREM%, REM% in BB vs. Cx.

BB: Blue blockers; CI: Confidence interval; Cx: Control; NREM: Non-rapid eye movement sleep; REM: Rapid eye movement sleep; SE: Sleep efficiency; SOL: Sleep onset latency; SWS: Slow wave sleep; TST: Total sleep time; WASO: Wake after sleep onset; Values are expressed as means ± SD unless otherwise indicated.

*Data for these outcomes were not reported in the published paper and were obtained by contacting study authors with a data request.

Ayaki *et al.* [25] observed that BB lenses vs. clear significantly reduced SOL and increased sleep efficiency (SE) but did not alter WASO in healthy adults exposed to luminous devices. In healthy adults, Knufinke *et al.* observed that BB lenses vs. clear did not affect TST, WASO, SOL, or SE. Also, in healthy adults, Ostrin *et al.* observed that TST was significantly longer after BB vs. baseline. They reported no differences in SE or SOL, but significantly lengthened WASO (56.08 ± 17.0 vs. 49.25 ± 18.43 minutes, *p* = 0.03) in treatment vs. baseline [26]. During the ambulatory portion of the study using accelerometry, van der Lely *et al.* [27] observed no differences in TST, SE, or fragmentation index in healthy adolescents. In the same study, there were no differences in any PSG sleep parameter when measured in the laboratory after exposure to LED light while wearing the lenses vs. control [27].

In adults with insomnia symptoms (Shechter *et al.*), BB lenses vs. clear significantly lengthened TST, but did not alter SOL, SE, or WASO [29]. In young adults with sleep complaints (Perez Algorta *et al.*), participants had no difference in sleep duration, SE, or awakenings in amber vs. control. In adults with DSPS (Esaki *et al.*), sleep onset time was significantly advanced after treatment compared to baseline, but wake time, TST, and SE were unchanged by treatment [32]. In individuals with bipolar disorder in the manic phase (Henriksen *et al.*), there was a significant effect of BB lenses vs. clear control on increasing SE and decreasing WASO, but TST and sleep fragmentation index were not different between treatments [34].

**Figure 4.**
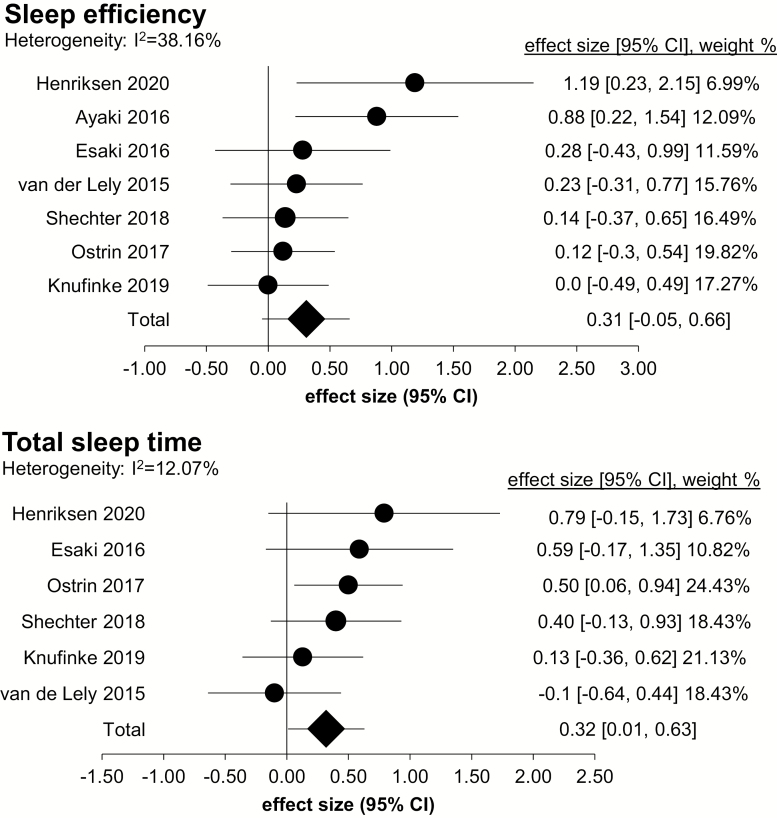
Forest plots of objective sleep outcomes. Comparisons are made between blue blocker lenses condition and control (or to pre-treatment baseline). Effect size, 95% confidence interval (CI), and weight of each study are shown on the right of each panel.

The forest plots for objective sleep parameters are shown in Figure 4. For SE, there was a small-to-medium overall combined effect size (Hedge’s *g* = 0.31; 95% CI: −0.05, 0.66; *k* = 7), with moderate heterogeneity (*I*^2^ = 38.16%). Subgroup analysis for SE showed that the combined effect size was relatively larger for “sleep and/or psychiatric patients” (Hedge’s *g* = 0.41; 95% CI: −0.84, 1.65, *k* = 3; *I*^2^ = 51.04%) than for “healthy participants” (Hedge’s *g* = 0.26; 95% CI: −0.31, 0.84, *k* = 4; *I*^2^ = 43.59%). For TST, there was a small-to-medium overall combined effect size (Hedge’s *g* = 0.32; 95% CI: 0.01, 0.63; *k* = 6), with low heterogeneity (*I*^2^ = 12.07%). Subgroup analysis for TST showed that the combined effect size was relatively larger for “sleep and/or psychiatric patients” (Hedge’s *g* = 0.52; 95% CI: 0.07, 0.98, *k* = 3; *I*^2^ = 0%) than for “healthy participants” (Hedge’s *g* = 0.21; 95% CI: −0.55, 0.97, *k* = 3; *I*^2^ = 38.84%). Due to excessive effect size heterogeneity (*I*^2^ > 75%), combined effect sizes were not calculated WASO (*I*^2^ = 77.54%, *k* = 6) and SOL (*I*^2^ = 81.96%, *k* = 5).

#### Self-report sleep measures.

Eight studies included self-report sleep measures as outcomes (Table 3) [23, 24, 26–29, 31, 33].

**Table 3.** Effects of interventions on self-report sleep

Author, year (ref #)	Sleep assessment method	Findings
Burkhart 2009 [28]	Sleep quality (1–10 Likert scale)	• Sleep quality rating was significantly higher in BB vs. Cx at 2-week endpoint, *p* < 0.001.
		o At baseline, sleep quality rating was worse in BB vs. Cx, *p* < 0.001.
Esaki 2017 [31]	Sleep quality (VAS) Sleep timing; SOL	• No difference in change-from-baseline in BB vs. Cx for bedtime, wake time, SOL, or sleep quality rating.
		o At baseline, sleep quality rating was worse in BB vs. Cx, *p* = 0.015.
Fargason 2013 [33]	PSQI; Post-sleep questionnaire; Sleep timing	• Global PSQI score improved in BB (4.54 ± 3.15) vs. baseline (11.15 ± 3.50), *p* < 0.05.
		• Improvements in all PSQI subscales in BB vs. baseline were significant: sleep quality: 0.62, *p* = 0.005, sleep latency: 1.46, *p* = 0.001, SE: 1.23, *p* = 0.001, sleep disturbance: 1.46, *p* < 0.001, sleep medication: 0.39, *p* = 0.096, daytime dysfunction: 0.77, *p* = 0.002 components (values are mean decrease in scores)
		• Number of nighttime awakenings significantly reduced in BB vs. baseline, *p* = 0.015.
Knufinke 2019 [23]	Sleep diary; Sleep quality (1–10 Likert scale)	• SOL was reduced in BB (12.0 ± 7.0 minutes) vs. Cx (19.0 ± 11.0 minutes); *p* = 0.005
		• Sleep quality was increased in BB (7.48 ± 0.76) vs. Cx (6.91 ± 1.02); *p* = 0.027
		• No differences in WASO, number of awakenings, or TST in BB vs. Cx.
Nagai 2019 [24]	PSQI	• Global PSQI score improved in BB (3.83 ± 0.28) vs. baseline (5.50 ± 0.61), *p* = 0.030.
		• TST and SE not different in BB vs. Cx.
Ostrin 2017 [26]	PSQI	• Global PSQI score improved in BB (3.0 ± 2.2) vs. baseline (5.6 ± 2.9), *p* < 0.01.
Shechter 2018 [29]	PIRS; Sleep diary; Sleep quality and soundness (1–7 Likert scale)	• PIRS was significantly improved in BB (72.64 ± 28.14) vs. Cx (88.93 ± 33.19), *p* = 0.023
		• TST was significantly longer in BB (399.33 ± 80.31 minutes) vs. Cx (347.11 ± 70.50), *p* < 0.01
		• No differences in SOL, SE, and WASO in BB vs. Cx.
		• Sleep quality rating (BB: 4.00 ± 1.39 vs. Cx: 3.31 ± 0.91, *p* = 0.032) and sleep soundness rating (BB: 4.34 ± 1.27 vs. Cx: 3.32 ± 1.15, *p* = 0.004) were significantly higher in BB vs. Cx.
van der Lely 2015 [27]	Leeds Sleep Evaluation Questionnaire	• No significant difference between BB and Cx.

BB: Blue blockers; Cx: Control; PIRS: Pittsburgh Insomnia Rating Scale; PSQI: Pittsburgh Sleep Quality Index; SE: Sleep efficiency; SOL: Sleep onset latency; TST: Total sleep time; VAS: Visual analog scale; WASO: Wake after sleep onset; Values are expressed as means ± SD unless otherwise indicated.

In healthy adults, Nagai *et al.* [24] observed that Pittsburgh Sleep Quality Index (PSQI) global score was significantly improved, while TST and SE based on self-report were not changed after treatment compared to baseline. Similarly, Ostrin *et al.* [26] also reported significantly improved PSQI scores after BB compared to pre-treatment baseline. In healthy adults, Knufinke *et al.* [23] observed that BB lenses vs. clear significantly reduced SOL and increased sleep quality, but did not affect other self-report measures including WASO, number of awakenings, and TST. In the van der Lely *et al.* [27] study, in healthy adolescents, BB lenses vs. clear did not affect sleep ratings based on the Leeds Sleep Evaluation Questionnaire.

In the Burkhart and Phelps study, on individuals with baseline sleep complaints, sleep quality rating based on a 1–10 Likert scale, with 0 being very poor and 10 being very good, was significantly higher in the BB lenses group compared to clear control after the treatment period [28]. Shechter *et al.* found that in individuals with insomnia symptoms, BB lenses vs. clear control caused significant improvements in Pittsburgh Insomnia Rating Scale scores [29]. Participants also reported significantly longer TST and higher ratings of sleep quality and soundness on a 1–7 Likert scale, but no differences in SOL, SE, or WASO [29].

In Esaki *et al.*, in individuals with MDD and sleep onset insomnia, there were no differences between groups in change-from-baseline in sleep quality (assessed via visual analog scale [VAS]) or self-reported bedtime, wake time, or SOL [31]. Fargason *et al.* [33] found that BB lenses improved self-reported sleep in individuals with ADHD and DSPS. The PSQI global score, as well as all subcomponents except sleep medications, were significantly improved after treatment compared to baseline. Participants also reported having significantly fewer nighttime awakenings after treatment [33].

**Figure 5.**
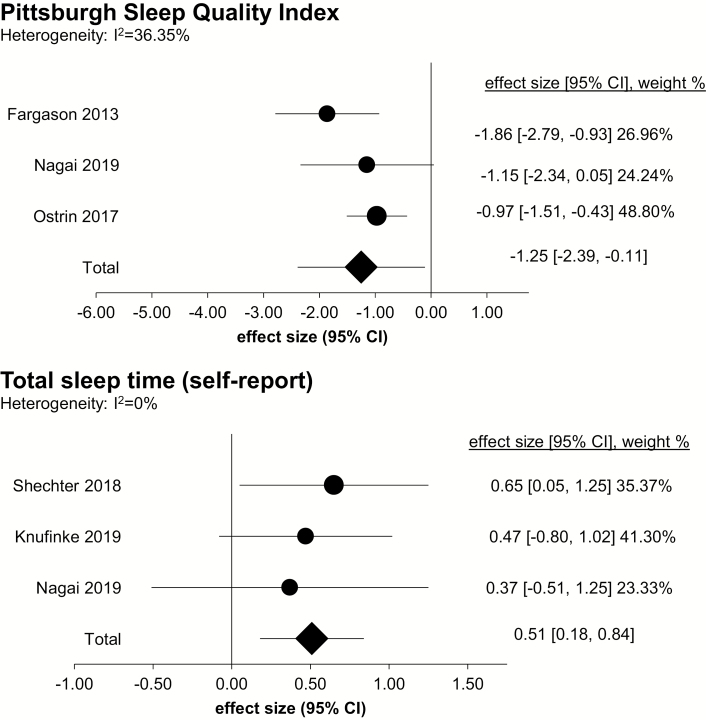
Forest plot of self-reported sleep outcomes. Comparisons are made between blue blocker lenses condition and control (or to pre-treatment baseline). Effect size, 95% confidence interval (CI), and weight of each study are shown on the right of the panel.

Heterogeneity in assessment method and the small number of studies precluded quantitative pooling of most self-report sleep outcomes. However, three studies included the PSQI and a self-report measure of sleep duration (Table 3; Figure 5). For PSQI, there was an overall combined effect size of large magnitude (Hedge’s *g* = −1.25; 95% CI: −2.39, −0.11; *k* = 3), with moderate heterogeneity (*I*^2^ = 36.35%). For TST, there was a combined effect size of medium magnitude (Hedge’s *g* = 0.51; 95% CI: 0.18, 0.84; *k* = 3), with low heterogeneity (*I*^2^ = 0%). Subgroup analyses were not conducted for PSQI and TST, since in each, there was one study including “sleep and/or psychiatric disorder participants” and two studies including “healthy participants.”

## Discussion

### Summary and considerations

In considering the 12 BB lenses for sleep studies individually, eight showed some positive effect [24–26, 28, 29, 32–34], and four showed no effect [23, 27, 30, 31] of the intervention. In terms of positive effects, the lenses were found in individual studies to improve self-report sleep quality ratings and parameters [24, 26, 28, 29, 33], sleep timing [26, 32], and accelerometer-derived measures of SOL [25], SE [25, 34], WASO [34], and TST [26, 29]. Using meta-analyses to determine combined effects sizes when sufficient data were available, there appears to be a small-to-moderate effect of the intervention on objective measures of SE and TST, as well as larger magnitude effects on self-reported sleep quality and sleep duration.

There is substantial heterogeneity in participant characteristics for these studies, which may contribute to inconsistencies and/or patterns in the observed effects. Not surprisingly, it appears that positive effects of the intervention were more commonly seen in studies that included individuals with sleep and/or psychiatric disorders, or in healthy individuals when exposed to a nighttime light-exposure challenge. For example, positive effects of the lenses were observed in those with insomnia/sleep difficulties [28, 29], bipolar disorder [34], DSPS [32], in those with ADHD and DSPS [33], and in healthy adults after exposure to portable luminous devices at night [25]. Indeed, when the meta-analyses for objective SE and sleep duration were conducted with a subgroup analysis, relatively larger combined effects sizes were seen for sleep and/or psychiatric patients compared to healthy patients. Healthy, good sleeping individuals may, therefore, garner relatively less benefit from using the lenses before habitual bedtime than other vulnerable groups.

We observed that the effects sizes in response to the BB intervention were different when sleep parameters were assessed subjectively and objectively. Specifically, effect sizes of self-reported sleep quality and duration were moderate-to-high, whereas small-to-moderate effect sizes were seen for accelerometry-derived measures of SE and duration. While we are unsure of the explanation, it is not uncommon for individuals, particularly those with sleep disturbances, to experience a discrepancy between their self-reported and objective sleep [35]. This may be relevant since individuals with sleep problems and/or psychiatric conditions were found to derive greater benefit than healthy individuals from the interventions described here. Furthermore, the largest effect sizes were seen for PSQI scores, a self-report measure of global sleep quality, as compared to other discrete measures of sleep. This may indicate a broader, non-selective improvement in response to the intervention. Nevertheless, given the subjective nature of many sleep complaints, a noted improvement in self-reported sleep quality and duration, as observed in several studies described here, may be of clinical benefit.

Duration of the intervention is a factor that may potentially play a role in the observed effects. There was considerable variation in the duration of intervention in the included studies, ranging from 2 days to 1 month. Information on duration of intervention is important to consider when designing future work on this topic, as well as in the application of these experimental findings to clinical settings. The treatment was applied for 1 or 2 weeks in individuals with insomnia symptoms [28, 29], and 2 or 3 weeks in individuals with DSPS [32, 33], each with some positive effect on sleep outcomes. Those developing non-pharmacologic therapies for patients with sleep or circadian disturbances (e.g. insomnia or DSPS) may, therefore, consider a treatment duration of around 2 weeks, although more work focused specifically on this should be done.

### Potential mechanistic pathways

Evening light exposure from normal ambient room lighting (<200 lux) causes reductions and delays in melatonin secretion [36], and evening light exposure of even lower levels during the 4 hours preceding bedtime is associated with prolonged SOL in the home setting [37]. The suppression and/or delay of nocturnal melatonin secretion by short-wavelength light exposure is a potential cause of sleep disturbance after the use of portable light-emitting electronic devices at night [8–13]. Pre-bedtime exposure to short-wavelength light can impact circadian physiology, sleep quality, and vigilance. Studies of short-wavelength light exposure in the hours before sleep from LED-backlit computers [8], eBooks [9], and tablets [10, 11] demonstrate that use of these common devices can reduce and/or delay melatonin secretion, as well as prolong SOL and reduce overnight rapid eye movement sleep [9]. Exposure to short-wavelength light also causes acute alerting effects, which can interfere with sleep initiation and maintenance [38], and reduce subjective and neurophysiologic measures of sleepiness in the evening [8, 9].

It is therefore hypothesized that the reduction in short-wavelength light exposure is the likely mode by which “blue blocking” lenses can benefit sleep. This is supported by studies which demonstrated that amber or orange-tinted lenses caused reduced melatonin suppression when participants were exposed to short-wavelength light during the night from computer monitors [39], tablets [10], or other self-luminous devices (computers, tablets, e-readers, televisions, and cell phones) [11].

Although reduced melatonin suppression and/or higher overnight melatonin secretion is the proposed mechanism of beneficial action, most studies did not assess this hormone. Only five of the studies included in the current systematic review reported melatonin levels in addition to sleep outcomes in response to the intervention [24–27, 32]. Ayaki *et al.*, who reported an improvement in objective sleep, also observed higher overnight melatonin secretion after participants wore BB vs. clear lenses while exposed to self-luminous devices. Ostrin *et al.* [26] observed significantly increased nighttime melatonin levels, and corresponding increases in objective sleep duration and self-reported sleep quality, in healthy individuals who wore lenses each night for 2 weeks. Esaki *et al.*, in individuals with DSPS who experienced significantly advanced sleep onset time, found that dim light melatonin onset (DLMO; saliva) was not statistically significantly advanced (by 78 minutes, *p* = 0.145) after treatment compared to baseline [32]. In the van der Lely *et al.* [27] study, despite no observed effects on nocturnal PSG sleep, melatonin levels were significantly higher in the BB vs. the clear lenses condition during the pre-sleep period of LED light exposure. Conversely, Nagai *et al.* [24] found no difference in overnight melatonin levels after 1 month of treatment compared to baseline, despite improved self-reported sleep quality.

Related to the above, overall levels and spectral composition of ambient light were not routinely assessed in these studies. This is important, since exposure to even moderate light levels, in addition to short-wavelength light, can acutely suppress melatonin [36]. Therefore, in the absence of detailed information about ambient light during the intervention period, the effects summarized here and the comparisons between studies should be interpreted with caution. Moreover, prior light history can impact the effects of acute light exposure on the circadian system [40]. Specifically, the effects of light exposure on melatonin suppression may be enhanced after both short- [41] and long-term [40] dim light exposure. Importantly, in the Ostrin *et al.* [26] study that showed a positive effect of BB on sleep, participants were found to receive a similar amount of daily light exposure (lux per day) during the baseline and treatment phases. Recent work suggests that the human circadian system can show high sensitivity to even relatively dim (<30 lux) evening light levels, and importantly, that sensitivity to evening light differs widely across individuals [42]. This suggests that inter-individual differences in light sensitivity may also factor into one’s response to this intervention. Future studies on the effects of BB lenses on sleep should assess ambient light levels and spectral composition, both acutely and habitually, as well as melatonin secretion, to separate physiologic and psychologically driven effects and more confidently infer a biological mechanism of action.

In addition to affecting melatonin and circadian pathways, short-wavelength light also has direct alerting effects, which may disturb sleep. In the van der Lely *et al.* [27] study, use of BB vs. clear lenses was associated with reduced objective alertness in the evening (psychomotor vigilance task) and increased self-reported sleepiness (Karolinska Sleepiness Scale). Therefore, while not routinely assessed in the studies described here, there is some evidence that this approach may impact vigilance to promote sleep initiation.

### Limitations of the studies

Some limitations in the studies conducted to date should be discussed in order to further qualify the reported effects. As mentioned above, there is considerable heterogeneity in terms of participants included in the studies. These include adolescents and adults, healthy individuals, and a range of pathophysiologic conditions (insomnia, DSPS, ADHD, MDD, bipolar). Considering the small number of studies, it is unclear how these and other specific participant characteristics may influence the effects of the intervention.

There are important considerations regarding the type of lenses that are used in the BB interventions. The included studies used brown, yellow, amber, or orange lenses. The degree of blockage of short-wavelength light and visible light, quantified in studies as BLT and VLT, respectively, differ substantially depending on the color of lenses. For example, some orange lenses achieve a near-complete blockage of “blue light” transmission (BLT: <2%), whereas some amber lenses achieve a more modest reduction of “blue light” (BLT: 35%). However, the orange lenses also result in a dimming of the overall light environment (VLT: 30–45%), whereas the amber lenses do not (VLT: 90%). Thus, in these BB lens studies, it is difficult to tease apart the effects of selective short-wavelength light reduction compared to an overall dimming of the ambient light environment on sleep outcomes. As described above, this is critical since light levels, and not just spectral composition, can affect sleep. The types of lenses should also be considered when designing interventions or recommendations for use. For example, amber lenses may be preferable for use in elderly individuals at risk for falls since they filter short-wavelength light but do not result in a dimming of light levels overall.

There was a wide variety of methods to assess sleep, which makes it difficult to compare findings across studies and likely contributes to discrepancies. These included wrist-accelerometry, PSG, standardized questionnaires (e.g. PSQI), sleep diaries, and other scales (VAS, Likert).

Adherence to the intervention is difficult to quantify, particularly in the studies that were conducted outside of the laboratory setting. Future work should aim to develop an objective method to monitor the use of BB lenses (e.g. temperature sensors on the frames).

Statistical power is likely a limitation for most of the included studies. The average sample sizes were *n* = 12 for the before-after studies, *n* = 13.2 for the crossover trials, and *n* = 9.5 per group in the parallel-arm trials (Table 1). These small sample sizes are particularly important for interpreting the negative findings of studies that may not have been powered to detect statistically significant differences in outcomes. Additionally, it should be noted that for many of the individual studies as well as some of the combined values (e.g. PSQI), the confidence intervals are relatively wide for Hedge’s *g*, therefore indicating that effect size estimates are likely imprecise.

### Conclusions

The findings of this systematic review and meta-analysis reveal that the “blue blocker” lenses approach to improving nocturnal sleep is a growing, though still relatively understudied, area of research. Clinical and methodological heterogeneity compounded by the small number of included trials made it difficult to conduct robust meta-analyses with more than 3–7 studies. There is mixed evidence on whether these approaches can benefit nocturnal sleep. The approach may be particularly useful for individuals with insomnia, bipolar disorder, DSPS, or ADHD, though less beneficial for healthy good sleepers. Considering the ubiquitousness of short-wavelength enriched light sources and the potential for widespread sleep impairment, more studies to examine the efficacy of this approach are warranted. Future well-controlled trials should include sufficiently large sample sizes, standardized outcomes, assessments of ambient light levels and spectral composition, and measurement of melatonin (for both direct- and circadian clock-dependent effects).

## Supplementary Material

zpaa002_suppl_Supplementary-Material
