# Supplementary material for: Interventions to reduce short-wavelength (“blue”) light exposure at night and their effects on sleep: A systematic review and meta-analysis
Source: Sleep Adv. 2020 Jun 4;1(1):zpaa002. doi: 10.1093/sleepadvances/zpaa002 (PMC10127364; doi:10.1093/sleepadvances/zpaa002)
Supplement: zpaa002_suppl_Supplementary-Material [file zpaa002_suppl_supplementary-material.docx]

**Title:** Interventions to reduce short-wavelength (“blue”) light exposure at night and their effects on sleep: A systematic review and meta-analysis

**Short title:** Blue light blocking and sleep

**Authors:** Ari Shechter^1,2,*^, Kristal A. Quispe^1^, Jennifer S. Mizhquiri Barbecho^1^, Cody Slater^3^, and Louise Falzon^4^

**Affiliations:** ^1^ Center for Behavioral Cardiovascular Health, Columbia University Irving Medical Center, New York, NY, USA

^2^ Sleep Center of Excellence, Columbia University Irving Medical Center, New York, NY, USA

^3^ Columbia University Vagelos College of Physicians and Surgeons, New York, NY, USA

^4^ Center for Personalized Health, Feinstein Institutes for Medical Research, Northwell Health, New York, NY, USA

*** Corresponding author:**

Ari Shechter, PhD

Department of Medicine

Columbia University Irving Medical Center

622 West 168^th^ Street, 9^th^ Floor

New York, NY 10032

Phone: 212-342-4487

Email: [as4874@cumc.columbia.edu](mailto:as4874@cumc.columbia.edu)

**SUPPLEMENTARY MATERIALS:**

SYSTEMATIC REVIEW SEARCH STRATEGY

**Blue-Light Blocking Systematic Review: Draft Search Strategy**

**Exp** = Explode. This indicates that there is at least one more specific MeSH term included

/ = Single MeSH heading

**$** = truncation. Picks up variable word endings e.g. sleep$ retrieves sleep, sleepiness, sleep-wake

**?** Replaces one character within a word. E.g. color or colour

**Adj** = adjacent to. Finds words next to each other or within a specified number of words of each other. E.g (blue adj2 light$ adj2 filter$).tw retrieves blue light spectrum filter or blue wavelength light filtering

**.tw.** = textwords. Words in the title or abstract

Database: Ovid MEDLINE(R)

Search Strategy:

--------------------------------------------------------------------------------

1 exp Sleep/ (72090)

2 exp Sleep Wake Disorders/ (78448)

3 exp chronobiology phenomena/ or circadian rhythm/ (96053)

4 exp Arousal/ (110930)

5 Melatonin/ (17888)

6 sleep$.tw. (136626)

7 Insomnia$.tw. (14883)

8 (alert$ or attention).tw. (315655)

9 circadian.tw. (40295)

10 melatonin.tw. (19720)

11 or/1-10 (638106)

12 Eyeglasses/ (7244)

13 light/ (96281)

14 Lighting/ (11261)

15 Eye Protective Devices/ (1718)

16 filtration/ (23178)

17 Color Therapy/ (80)

18 (blue adj3 (block$ or reduc$ or wavelength$)).tw. (2075)

19 ((amber or orange or espresso or tinted) adj2 (lense$ or glasses or spectacle$)).tw. (207)

20 ((colo?r or dark$) adj therap$).tw. (37)

21 (blue adj2 light$ adj2 filter$).tw. (150)

22 (blue adj3 filter$).tw. (347)

23 exp microcomputers/ (19222)

24 microcomputers/ or minicomputers/ (15061)

25 (computer$ or tablet$ or electronic device$ or phone$ or smartphone$ or screen or screens or ipad$ or iphone$).tw. (408059)

26 or/23-25 (418411)

27 (setting$ or mode$ or night or dim or dimmed or dark or shift).tw. (3433942)

28 26 and 27 (112187)

29 or/12-22,28 (250093)

30 11 and 29 (18608)

31 randomized controlled trial.pt. (462326)

32 controlled clinical trial.pt. (92434)

33 randomized.ab. (362128)

34 placebo.ab. (173143)

35 drug therapy.fs. (2024674)

36 randomly.ab. (250817)

37 trial.ab. (375494)

38 groups.ab. (1566648)

39 or/31-38 (3904286)

40 non-randomized controlled trials as topic/ (359)

41 interrupted time series analysis/ (435)

42 controlled before-after studies/ (329)

43 ((controlled or control group$ or (before adj5 after) or (pre adj5 post) or (pretest or pre test)) and (posttest or post test)).tw. (11445)

44 (quasiexperiment$ or quasi experiment$).tw. (8667)

45 (time series or time point$ or repeated measur$).tw. (134729)

46 or/40-45 (152711)

47 39 or 46 (3998131)

48 exp animals/ not humans.sh. (4466015)

49 47 not 48 (3399887)

50 30 and 49 (2522)
